# Protein Interaction Networks—More Than Mere Modules

**DOI:** 10.1371/journal.pcbi.1000659

**Published:** 2010-01-29

**Authors:** Stefan Pinkert, Jörg Schultz, Jörg Reichardt

**Affiliations:** 1 Department of Bioinformatics, Biocenter, University of Würzburg, Würzburg, Germany; 2 Department of Cellular Biochemistry, Max Planck Institute of Biochemistry, Martinsried, Germany; 3 Institute for Theoretical Physics and Astrophysics, University of Würzburg, Würzburg, Germany; 4 Complexity Sciences Center, University of California at Davis, Davis, California, United States of America; Weizmann Institute of Science, Israel

## Abstract

It is widely believed that the modular organization of cellular function is reflected in a modular structure of molecular networks. A common view is that a “module” in a network is a cohesively linked group of nodes, densely connected internally and sparsely interacting with the rest of the network. Many algorithms try to identify functional modules in protein-interaction networks (PIN) by searching for such cohesive groups of proteins. Here, we present an alternative approach independent of any prior definition of what actually constitutes a “module”. In a self-consistent manner, proteins are grouped into “functional roles” if they interact in similar ways with other proteins according to their functional roles. Such grouping may well result in cohesive modules again, but only if the network structure actually supports this. We applied our method to the PIN from the Human Protein Reference Database (HPRD) and found that a representation of the network in terms of cohesive modules, at least on a global scale, does not optimally represent the network's structure because it focuses on finding independent groups of proteins. In contrast, a decomposition into functional roles is able to depict the structure much better as it also takes into account the interdependencies between roles and even allows groupings based on the absence of interactions between proteins in the same functional role. This, for example, is the case for transmembrane proteins, which could never be recognized as a cohesive group of nodes in a PIN. When mapping experimental methods onto the groups, we identified profound differences in the coverage suggesting that our method is able to capture experimental bias in the data, too. For example yeast-two-hybrid data were highly overrepresented in one particular group. Thus, there is more structure in protein-interaction networks than cohesive modules alone and we believe this finding can significantly improve automated function prediction algorithms.

## Author Summary

Cellular function is widely believed to be organized in a modular fashion. On all scales and at all levels of complexity, relatively independent sub-units perform relatively independent sub-tasks. This functional modularity must be reflected in the topology of molecular networks. But how a functional module should be represented in an interaction network is an open question. On a small scale, one can identify a protein-complex as a module in protein-interaction networks (PIN), *i.e.*, modules are understood as densely linked (interacting) groups of proteins, that are only sparsely interacting with the rest of the network. In this contribution, we show that extrapolating this concept of cohesively linked clusters of proteins as modules to the scale of the entire PIN inevitably misses important and functionally relevant structure inherent in the network. As an alternative, we introduce a novel way of decomposing a network into functional roles and show that this represents network structure and function more efficiently. This finding should have a profound impact on all module assisted methods of protein function prediction and should shed new light on how functional modules can be represented in molecular interaction networks in general.

## Introduction

Biological function is believed to be organized in a modular and hierarchical fashion [1]. Genes make proteins, proteins form cells, cells form organs, organs form organisms, organisms form populations and populations form ecosystems. While the higher levels of this hierarchy are well understood, and the genetic code has been deciphered, the unraveling of the inner workings of the proteome poses one of the greatest challenges in the post-genomic era [2]. The development of high-throughput experimental techniques for the delineation of protein-protein interactions as well as modern data warehousing technologies to make data available and searchable are key steps towards understanding the architecture and eventually function of the cellular network. These data now allow for searching for functional modules within these networks by computational approaches and for putatively assigning protein function.

A recent review by Sharan *et al.* [2] surveys the current methods of network based prediction methods for protein function. Proteins must interact to function. Hence, we can expect protein function to be encoded in a protein interaction network. The basic underlying assumption of all methods of automated functional annotation is that pairwise interaction is a strong indication for common function.

Sharan *et al.* differentiate two basic approaches of network based function prediction: “direct methods”, which can be seen as local methods applying a “guilt-by-association” principle [3] to immediate or second neighbors in the network, and “module assisted” methods which first cluster the network into modules according to some definition and then annotate proteins inside a module based on known annotations of other proteins in the module. So instead of “guilt-by-association”, one could speak of “kin-liability”. The latter approach to function prediction necessitates a concept of what is to be considered a module in a network. Most researchers consider cohesive sets of proteins which are highly connected internally, but only sparsely with the rest of the network [4]–[14]. Such methods have yielded considerable success at the level of very small scale modules and in particular protein complexes.

Is the concept of a module as a group of cohesively interacting proteins also useful on larger scales? Some researchers have argued that modularity in this sense is a universal principle such that small cohesive modules combine to form larger cohesive entities in a nested hierarchy [15],[16]. But is this view really adequate to describe the architecture of protein interactions? Recently, Wang and Zhang [17] questioned whether cohesive clusters in protein interaction networks carry biological information at all and suggested a simple network growth model based on gene duplication which would produce the observed structural cohesiveness as “an evolutionary byproduct without biological significance”. We will not go as far as questioning the content of biological information in the network structure but rather argue against the model of a cohesively linked group of nodes in a network as an adequate proxy for a functional module on all scales of the network.

Consider, as first example, protein complexes. Indeed, they consist of proteins working together and experimentally isolated together. Only the large scale analysis of protein complexes [18],[19] revealed that they are more dynamic than previously assumed. Many proteins can not only be found in a single, but in a multitude of complexes. The information about proteins connecting complexes will be lost when searching only for cohesively interacting groups of proteins. As a second example, consider transmembrane proteins, like receptors in signal transduction cascades. They tend to interact with many different cytoplasmic proteins as well as with their extra-cellular ligands. Still, only rarely do different transmembrane receptors interact with each other. Thus, the functional class of transmembrane receptors will not be identified when looking for cohesive modules.

Here, we ask whether such features, which are not discovered by algorithms searching for cohesive modules, are also present in the overall structure of the cellular network. If this is the case, methods searching only for cohesive modules would not be able to identify them. We group proteins self-consistently into *functional roles* if they interact in similar ways with other proteins according to their functional roles. Such a role may well be a cohesive module, meaning that proteins in this class predominantly interact with other proteins of this class, but it does not have to. In other words, we do not impose a structure of cohesive modules on the network in our analysis but rather find the structural representation that is best supported by the data. Using the abstraction of a functional role, we generate an “image graph” of the original network which depicts only the predominant interactions among classes of proteins, thus allowing a bird's-eye view of the network.

In the case of a protein interaction network studied here, we found sound evidence that cohesive modules on a global scale do not adequately represent the network's global structure. We found cohesive groups of proteins acting as intermediates and specifically connecting other groups of proteins. Furthermore, we even identified groups of proteins which are only sparsely connected within themselves, but with similar patterns of interaction to other proteins. Thus, approaches searching only for cohesive modules which are sparsely connected to the rest of the network might not be sufficient to represent all characteristics of cellular networks. Our findings suggest that hierarchical modularity as nested, cohesively interacting groups of proteins has to be reconsidered as a universal organizing principle.

### 

#### Functional role decomposition and image graphs

**Figure 1:**
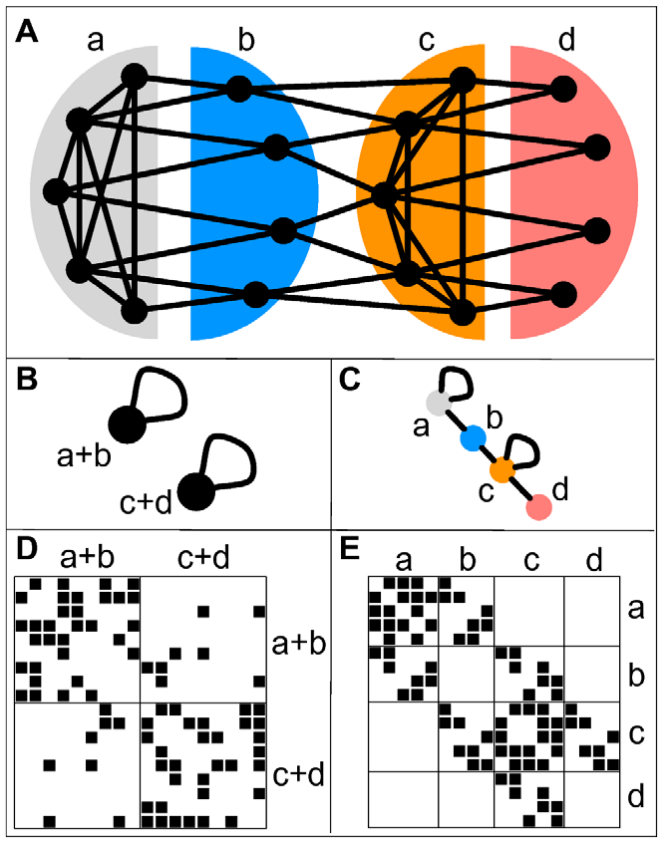
An example network and possible image graphs. A A simple example network of nodes of 4 different types identified by their structural position. Nodes of types 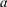 and 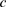 are densely connected among themselves. The nodes of type 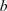 have connections to both nodes of types 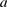 and 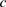, but not among themselves, i.e. they mediate between types 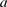 and 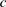. The nodes of type 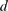 only have connections to nodes of type 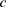, but not among each other, i.e. they form a periphery to type 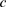 nodes. B and C Two possible image graphs for the functional understanding of this network show the connections among groups of nodes. A typical network clustering will aggregate nodes into clusters densely connected internally but only sparsely connected to the rest, as depicted in the left image graph. This will result in grouping nodes of types 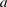 and 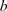 together and nodes of type 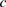 and 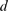 together. Because of aggregating nodes into cohesive groups, any such algorithm will never recognize nodes of type 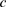 and 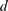 as different and hence miss essential part of the network's structure. On the opposite, the right image graph correctly captures the network structure of the 4 different types as the 4 different nodes in the image graph. D and E The adjacency matrices of our example network with rows and columns ordered according to the two decompositions shown above. A black square in position 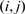 indicates the existence of a link connecting node 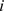 with node 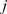. Rows and columns are ordered such that nodes in the same group are adjacent. The internal order of the nodes in the groups is random. Each block in the matrix corresponds to a possible edge in the image graph. The left matrix shows the adjacency matrix for the output of a typical clustering algorithm which groups nodes of type 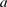 and 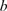, as well as 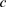 and 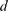 together. Clearly, we see dense blocks along the diagonal and sparse blocks on the off-diagonal of the matrix as expected. The right matrix depicts the adjacency matrix with rows and columns according to the actual types of the nodes. All empty blocks in this matrix correspond to a missing edge in the image graph and all populated blocks are represented by an edge in the image graph. We see that for this network, the image graph perfectly captures the structure of the network.

In which cases does a clustering of a network into cohesive modules not reflect its original architecture? Consider the toy network in figure 1 A. There are four known types of proteins in this network. Type 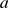 may represents some biological process involving five proteins connected to four proteins of type 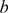. These are linked to another biological process 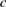 which involves five further proteins which finally are linked to four proteins of type 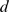. Not all nodes of the same type necessarily share the same set of neighbors. Some nodes of the same type do not have any neighbors in common with nodes of their type or have more neighbors in common with nodes of a different type. This shows that in this hypothetical example, direct methods of functional annotations may be limited in their accuracy.

Clustering the network into cohesive modules cannot capture the full structure of the network. The nodes of type B will never be recognized as a proper cluster, because they are not connected internally at all.

The structure of the example network can, however, be perfectly captured by a simple image graph with 4 nodes (Fig. 1 C). The nodes in an image graph correspond to the types of nodes in the network. Nodes of type 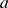 are connected to other nodes of type 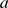 and to nodes of type 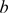. Nodes of type 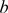 have connections to nodes of types 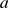 and 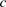 and so forth. The concept of defining types of nodes by their relation to other types of nodes is known as “regular equivalence” in the social sciences [20],[21]. Structure recognition in networks can then be seen as finding the best fitting image graph for a network. In this context, clustering into functional modules means representing the network by an image graph consisting of isolated, self-linking nodes. Once an assignment of nodes into classes is obtained, the rows and columns of the incidence matrix can be reordered such that rows and columns corresponding to nodes in the same class are adjacent (Fig. 1 D and E). The ordering of rows and columns representing nodes in the same class is random. This leads to a characteristic structure with dense blocks in the adjacency matrix corresponding to the links in the image graph and sparse or zero blocks corresponding to the links absent in the image graph. Structure recognition in networks is therefore also called “block modeling” and together with the concepts of structural and regular equivalence has a long history in the social sciences [22],[23]. In our further discussion, we will denote image graphs that consist only of isolated, self-linked nodes as in figure 1 B, “diagonal image graphs” due to the block structure along the diagonal in the adjacency matrix that they induce. Accordingly, we will call all other image graphs “non-diagonal image graphs”.

#### Calculation

But how do we find the best fitting image graph? There are two aspects to this question. On one hand, there is the topology of the image graph itself represented by its 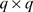 adjacency matrix 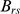, and on the other hand, there is the mapping 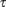 of the 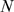 nodes of the network to the 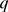 types of nodes such that the mismatch between network and image graph is minimal.

Let us focus on the latter aspect and suppose we have already the adjacency matrix 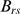 of our image graph together with the 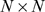 adjacency matrix 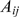 of our network. Let 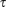 be the mapping of the 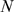 nodes to the 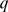 different types, such that 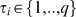 for all 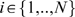. To optimize the mapping 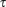 we minimize the following error function:
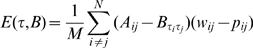
(1)

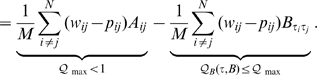
(2)in which 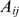 is the 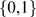 adjacency matrix of the network under study. 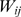 denotes the weight given to an edge between nodes 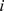 and 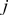. If an edge is absent in the network, 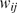 is naturally zero. As before 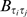 is the image graph and 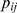 is a penalty term discussed below. The normalization constant 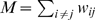 is used to bound the error by one. This error function gives a weight proportional to 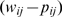 to errors made on fitting the edges in the network and a weight of 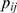 to errors made on fitting the absent edges in the network. The penalty term 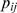 is chosen such that the total error weight on all edges in the network is equal to the total error weight on all absent edges in the network:
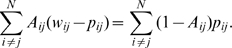
(3)This can be easily achieved by setting 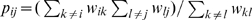.

The first term of equation (2) neither depends on the mapping of nodes to types 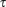 nor on the image graph 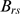. It can be interpreted as the maximum value 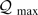 of a quality function 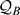 measuring the fit of the image graph to the network which would be obtained for a perfect fit with zero error, *i.e.* 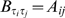 for all 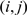. The second term in (2) then corresponds to the quality of the *actual* fit for the given image graph and mapping. The error is simply the difference between the best and any sub-optimal fit. Minimizing 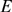 and maximizing 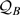 are equivalent.

Note that perfect fit or zero error can also be achieved if 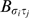 represents the classes of structurally equivalent nodes in the network. This simply means that all nodes of the network which have exactly the same set of interaction partners are mapped onto the same node of the image graph. When ordering the rows and columns of the adjacency matrix according to this partition of nodes into classes, only zero and full blocks are present.

How do we interpret the values of 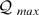 and 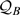, respectively? For a sparse network in which the average number of interaction partners per node is very small compared to the total number of nodes in the network, the value of 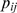 will be very small in comparison to 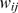 and, hence, 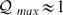. Since 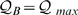 is only achieved if 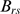 exactly mimics the network, we can interpret the ratio of the two values as an indication of how closely the image graph resembles the network. 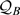 generally grows non-linearly with the number of classes, resp. the size of 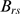.

If we assume a diagonal image graph 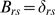 we recover in 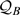 of equation (2) a popular quality function for graph clustering known as Newman modularity [17],[24],[25]:
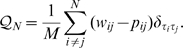
(4)We can directly compare the fit of different given image graphs to one network by the maximum score 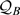 than can be obtained by optimizing the mapping 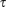 of nodes in the network to the classes represented as nodes in that image graph.

The overall optimal image graph with a given number of nodes 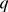 and the optimal assignment 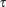 into the 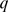 classes can be found directly by searching for the assignment 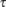 which maximizes [26],
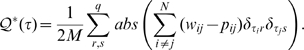
(5)The image graph which allows the highest value of 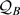 among all possible image graphs with this number of classes can be read off from the assignment 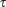 that maximizes (5). It must be such that 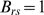, if the argument in the absolute value in (5) is strictly positive, and zero otherwise.

The parameter 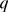 in (5) only represents the maximum possible number of classes. Any optimization routine is free to leave one or more of the 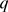 allowed classes unused. Provided that the optimization routine is not caught in local optima, 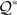 thus cannot decrease with increasing 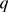. We found it strictly increasing with 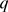 in all cases as 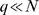 in the this work as the additional degrees of freedom always lead to an improved fit score.

Optimization of (5) is, just as optimization of (4) [28], NP-complete. We used simulated annealing [29] with local updates for the mapping of nodes into classes for the optimization. One such local update takes 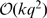 operations for (5) and 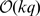 operations for (4) where 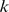 is the number of interaction partners of the node to update. These local updates have to be performed for all nodes in the network introducing a linear dependence on the size of the network into these estimates. Very slow cooling, however, may be required to escape local optima. Optimization by simulated annealing is, in principle, guaranteed to reach a globally optimal solution only for infinitely slow cooling schedules. In all our analyses, we have used only the best scoring solutions we found from multiple runs and are confident to have found solutions very close to the global optimum.

#### Comparison to alternative methods and benchmarks

One can view 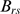 as a lossy compression of the original network with the goal to represent as many interactions as possible by edges in the image graph and as many missing interactions as possible by missing interactions in the image graph. The more pronounced a block structure is the adjacency matrix of the network, the better the compression will be. A number of recent publications deal with the detection of block structures in networks. Among them are the mixture model approach by Newman and Leicht [30] and a module detection method based on a compression algorithm and the minimum description length principle due to Rosvall and Bergstrom [31]. Additionally, we include a non-negative matrix factorization similar to that proposed by Lee and Seung [32], but instead of factorizing into two matrices, we use a symmetric tri-factorization as proposed by Ding *et al.* [33] which allows for a direct assignment of nodes into classes from the factorization. See the methods section for details.

Using a set of test networks with a known block structure, we compare these methods by measuring their performance as we increase the noise level. These networks have 128 nodes which are members, by design, of 4 different classes. Two of these classes are cohesive modules and two form a bi-partite structure with links running mainly between nodes in different classes. This setting was already used in Ref. [30] for benchmarking. The average number of neighbors per node is kept fixed at 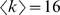. We can tune the difficulty of the structure detection task by the percentage of edges that do not adhere to the designed block structure, *i.e.* the noise level. For example, at a noise level of 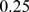, every node has, on average, 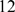 out of 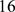 connections conforming to the designed block model, and 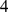 out of 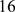 connections not conforming to the designed block model. This set of test-networks is a particularly difficult one since all nodes have the same degree and all nodes are in classes which have exactly one link in the image graph. This leads to all dense (sparse) blocks being equally dense (sparse) in the adjacency matrix and this symmetry makes structure detection particularly hard. We measure the accuracy of structure detection using the normalized mutual information (NMI) [34] between the designed classification and the one obtained by the different algorithms (see the methods section for details).

This particular set of benchmarks also shows a situation where the approach put forward by Guimera *et al.* in Ref. [25] fails. There, the authors first cluster the network into cohesive modules and then quantified the error in this approach as a “participation coefficient”, *i.e.* the fraction of links each node has connecting to other members of its own cluster. This participation coefficient is then used to differentiate proteins assigned to the same cohesive module. Applying this methodology to the set of test networks described above will fail to detect the bi-partite structure as the two groups of proteins will be recognized as one large cohesive cluster in which every node has the same high participation coefficient.

**Figure 2:**
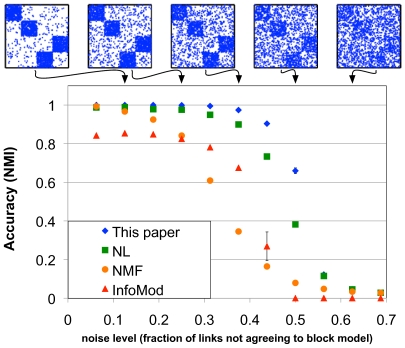
Benchmark on networks with known role structure. We compare our method with a mixture model approach by Newman and Leicht (NL) which employs a maximum likelihood approach [30], a non-negative matrix factorization (NMF) minimizing the Kullback-Leibler-divergence between data and estimated factors [32], and an approach based on minimum description length by Rosvall and Bergstrom (InfoMod) [31]. The adjacency matrices show typical realizations of the test networks with rows and columns ordered according to the designed classes. Accuracy is measured in terms of normalized mutual information (NMI) between the designed assignment of nodes into classes and the classification inferred by the algorithms. Clearly, our approach outperforms the alternatives, in particular for high noise levels.

Figure 2 shows the results of the benchmarks. Clearly, the the method proposed here outperforms the alternatives and gives a particularly large advantage for large noise levels.

All of the above approaches follow a top-down strategy, assigning all nodes in the network to one of generally only a few classes called modules or functional roles. This approach aims at the macro and meso-scale structure of the network. It is worth contrasting these approaches with those following a bottom-up strategy, such as the Power Graph method by Royer *et al.* [35]. This approach presents a loss-less compression of the network by collapsing cliques into “power nodes” and bi-cliques into “power edges”. It attempts to reduce the visual complexity of a network and as such, must then proceed in a hierarchical manner, since the typical clique and hence power node cannot contain more nodes than the the typical number of neighbors. The same applies for bi-cliques. So in very large networks, the clarity that is gained from collapsing parts of the network into power nodes and power edges is partly lost in the hierarchy of the recursive application. Also, since most of the currently available data on protein interaction is noisy and incomplete and contains false positive interactions, we find a lossy compression more adequate for the analysis of the large scale structure of the network.

Methodologically, a method similar to ours was also presented by Qi *et al.* [36], though these authors focus on genetic interaction in yeast. Qi *et al.* however, divide the set of all interaction partners into a set of “query” and “library” genes and restrict themselves to the analysis of putative functional similarity among the query genes due to similarity in interaction with the set of library genes. In contrast, our method aims at dividing the entire corpus of interaction partners self-consistently into functional classes.

## Results

### 

#### Network analysis

**Figure 3:**
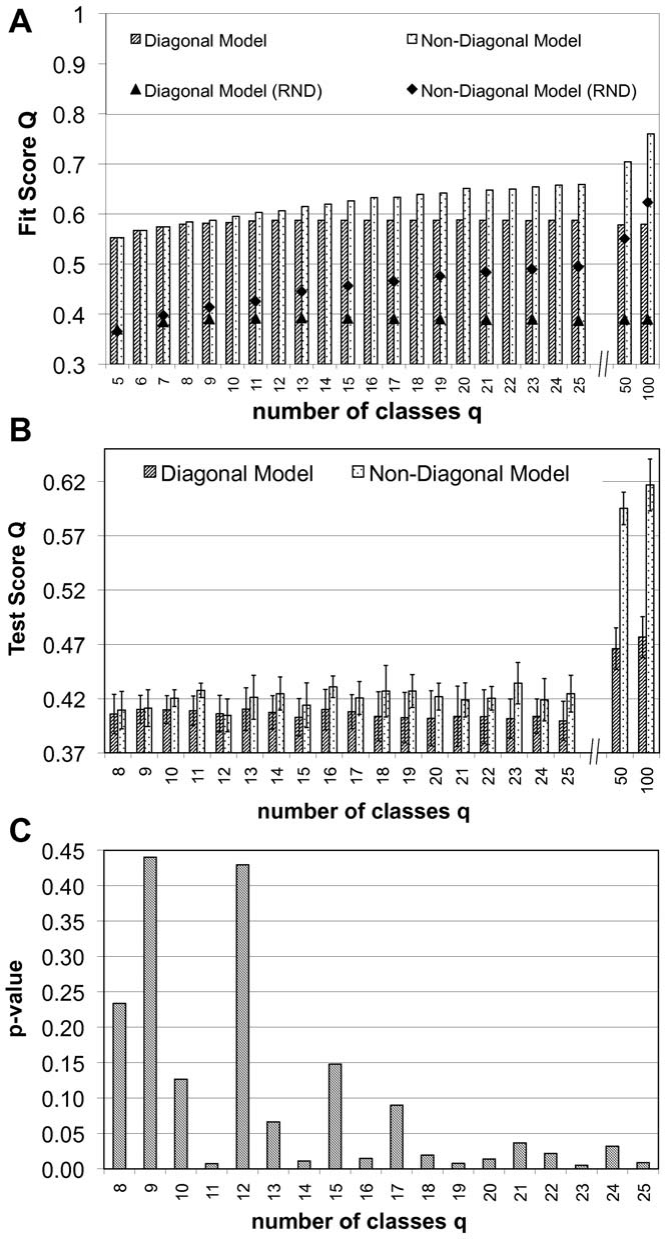
Fit scores and generalization error. A Comparison of highest fit scores 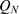 (4) and 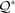 (5) for the full HPRD dataset with 32,331 interactions. Aggregating nodes into cohesive groups (diagonal image graphs) cannot improve the score beyond a certain limit, while non-diagonal image graphs are able to capture more and more structure as the image graph gets larger and larger. For comparison, the analysis was repeated on a randomized (RND) version of the original network. Standard deviation is smaller than symbol size. The fit scores we obtain on the real data show that the structure we find is far from random. B After removing a test-set of links from the network, we optimized the assignment of nodes into classes according to (2) using only the remaining links and keeping the image graphs fixed to those found in the runs that lead to figure A. With the assignment of nodes into classes for this training set of links, we computed the score on the test set of links. The figure shows average and standard deviation over 10 repetitions of this experiment. C p-values of Student's t-test for a statistically significant difference in the means of the test scores of panel B. For higher numbers of classes and thus larger differences in the fit scores of diagonal and non-diagonal image graphs, all differences become significant at the 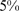 level.

Using the quality function introduced above, we analyzed the HPRD protein interaction network containing more than 8,500 nodes. We considered the entire network and optimized 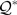 from (5) - thus finding optimal image graphs and assignments of nodes into classes. With increasing number of classes 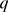, the fit between the actual network and the image graphs becomes better (Fig. 3 A). The maximum fit score was equal to 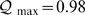. Therefore, even with a very small number of classes, already 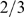 of the maximum fit score to the network was achieved. Restricting the image graphs to a diagonal form 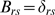 also limited the fit score. The maximum of 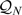 for a diagonal image graph was reached at 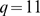 and further addition of classes did not increase this value significantly any more. For 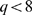 the fit scores for diagonal and non-diagonal image graphs were equal because for less than 8 classes the best image graphs were in fact diagonal. Only beyond this point did the additional degrees of freedom of the non-diagonal image graphs allow better scores.

We repeated the analysis on a set of networks generated by randomly rewiring the original network (see methods for details), but keeping the number of interactions and their respective type constant at each node. The fit scores we found for these randomized networks were much lower than what we found on the original data, clearly showing the the structure we find is genuine. See figure 3 A.

We now ask whether the additional degrees of freedom in the non-diagonal image graph actually convey information or only led to overfitting. We therefore divided the 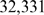 links of the network into a test- and a training-set. Using the optimal image graphs obtained on the full data set and diagonal image graphs for comparison, we optimized 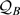 from (2) on the training-set of links and with the resulting mapping of nodes into classes calculated the fit score 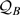 on the test-set. The fit score on the training-set of links (data not shown) was close to the full data set. We fixed the non-diagonal image graphs because the comparison is made to diagonal image graphs which were unaltered, too.

Both diagonal and non-diagonal image graphs showed overfitting to some extent. The score on the test set is lower than on the training set (Fig. 3 B). However, with increasing number of classes and thus increasing difference in fit-score over diagonal image graphs, the non-diagonal image graphs also scored better on the test-set, *i.e.* the increased fit value also generalized! Panel C of figure 3 shows the corresponding p-values of a Student's t-test. The non-diagonal image graphs do contain more information about the network structure than the diagonal image graphs.

The choice of the size of the test-set is a compromise between the need for a large test-set leading to a small variance in the test-score and not disturbing the network structure too drastically. For 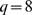 to 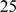 classes, we used a test-set of 1000 randomly chosen links from the network. This corresponds to 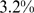 and represents a non-negligible disturbance of the system. If we assigned nodes into 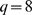 equal sized classes, we expect approximately 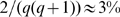 of all links in one block. So above this point, the test set we removed was more than the typical number of links in a block. Also, consider the average degree of 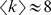 interactions per protein in the network. Removing a single link means removing on average 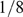 of the neighborhood of the nodes connected by this edge. For a test set of 1,000 edges, this could happen to 2,000 different nodes and thus to almost one quarter of all nodes which is similar to the typical 80/20 division used in tests of supervised learning algorithms. For 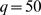 and 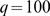, we used a test-set of 100 edges, as the test-set of 1000 edges proved to be too large a disturbance to the system.

#### Comparison of annotation quality

Now that we have shown that non-diagonal image graphs are better suited to represent the global structure of the HPRD PIN, we ask whether they also better represent biology? To answer this question we performed a GeneOntology (GO) enrichment analysis for all clusters using the “Ontologizer” software by Grossmann *et al.* [37]. We chose this software because it features a statistical control against the effects introduced by the structure of the GeneOntology, *i.e.* the parent-child relations of its terms. We tested each class of proteins found for enrichment of a particular GO annotation with the rest of the network as control group.

The comparison of the annotation quality of the diagonal models versus the non-diagonal models is difficult, as both methods focus on different aspects of the network structure. While the non-diagonal image graphs try to capture any link pattern, the diagonal image graphs try to capture maximally cohesive groups only. Both methods, however, can lead to a partition of the network into groups which are significantly enriched in GO terms. Another problem is that we cannot expect that the structure of the PIN is just another representation of the GeneOntology which, after all, was designed as a controlled vocabulary to describe gene products and has a very particular structure of terms of its own.

**Figure 4:**
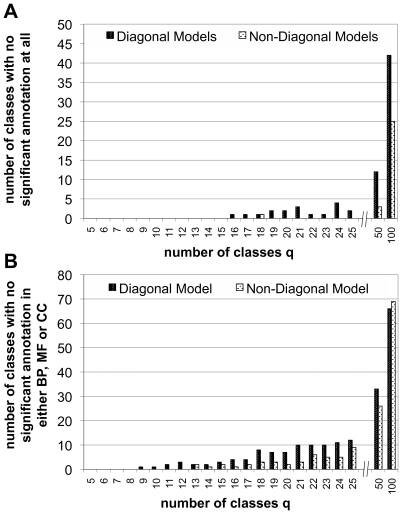
Number of classes *not* enriched in GO-terms with high significance. A Number of classes *not* significantly enriched below the 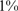 level after Bonferroni-correction in any of the GO categories biological process, molecular function or cellular compartment. B Number of classes *not* significantly enriched below the 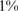 level after Bonferroni correction in at least one the GO categories biological process (BP), molecular function (MF) or cellular compartment (CC). Note the different scales. Clearly, the non-diagonal models consistently produce a lower number of classes which are not enriched in functional annotation. This can be seen as an indication that the non-diagonal models not only represent the network better, but the inferred groups also correspond better to known biology.

Due to these considerations, we compare the two approaches in a very simple manner by the number of classes of proteins they detect which *do not* have significant enrichment of GO-terms. We count a class of proteins as enriched, if at least one GO term is enriched at the one percent significance level after Bonferroni-correction for the number of GO terms we test. Panel A of 4 shows the number of classes which lack enrichment in all three basic categories of the GeneOntology (biological process, molecular function and cellular component). Panel B of figure 4 shows the number of classes which lack enrichment in at least one of the basic GeneOntology categories. For both diagonal and non-diagonal models, we find the number of classes without highly significant annotation increasing with the total number of classes allowed for. This has several reasons: First, not all proteins are annotated or are annotated with only very generic terms. Second, for higher numbers of classes, classes typically become smaller which together with a nonspecific annotation renders them not significant. The third effect is that as more classes are allowed for, models, especially diagonal, tend to separate densely connected, and most likely well researched and hence more specifically annotated, cores from a rather sparse periphery which then does not give statistically significant enrichment.

Nevertheless, one trend is consistently observed: the non-diagonal models produce fewer classes of proteins without annotation, both when looking at the number of classes without any enrichment, and when looking at the classes with missing enrichment in at least one of the basic GO categories. We take this as a clear indication that the non-diagonal block models are not only able to better represent the network, but also the known biological functional annotation. Notwithstanding, it may well be that a particular GO-term is enriched in a class detected by the diagonal model with a lower p-value. But the general trend is that the non-diagonal models produce an assignment of all nodes in the network into classes that is more consistent with the GeneOntology, because there are fewer classes without enrichment.

The complete GO annotation of all clusters of classifications into 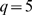 to 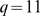, 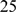, 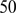 and 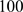 classes is available at http://domains.bioapps.biozentrum.uni-wuerzburg.de/ppi/. The careful reader will observe that the size of the classes of proteins varies widely when allowing for up to 50 or 100 classes. Especially for diagonal models, some classes contain only a few proteins, while others contain a few hundred. One might argue that mainly the small classes are those without significant annotation and therefore ask for a better partition with more balanced sizes. This is possible simply by increasing the penalty term 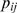 in equation (4) as proposed in [38]. However, our goal here is to compare diagonal and non-diagonal models for the organization of a PIN on equal footing. Hence, we should keep the penalty term in the quality function for the diagonal and non-diagonal models equal. Also, having more balanced sizes would necessarily mean splitting some of the larger groups which are now significantly enriched and hence might lose this enrichment in the process. Furthermore, when looking at the models with a smaller number of classes, we find that it is by no means only the smallest classes that turn out to be not enriched in any GO-term.

#### Examples of annotation and biological interpretation

**Figure 5:**
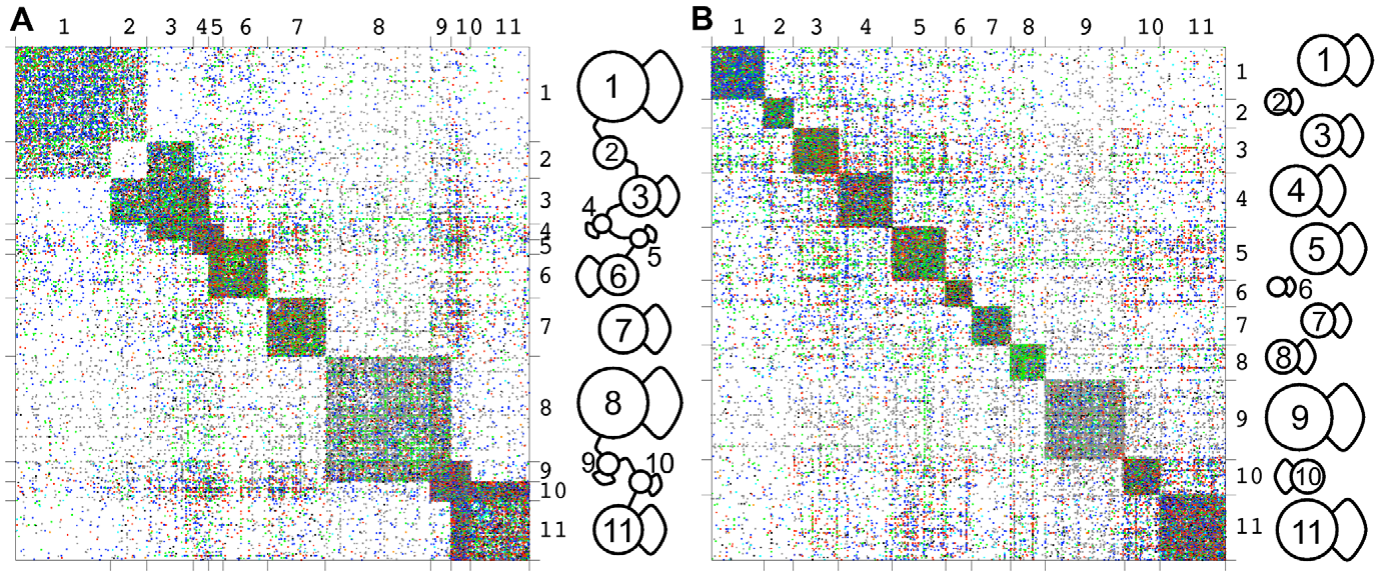
Comparison of block assignment. For 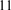 classes, we show the adjacency matrix of the HPRD protein interaction network with rows and column ordered to show non-diagonal (A) and diagonal (B) block structure plus the corresponding image graphs for diagonal block models and non-diagonal block models. Note how the non-diagonal models allow to capture overlap between cohesive blocks but also to detect groups of nodes which are non-cohesive but have similar connection patterns to other classes of proteins. The color of the links codes the experiment type: *Y2H*: grey, *in-vitro*: blue, *in-vitro*+*Y2H*: turquoise, *in-vivo*: green, *in-vivo*+*Y2H*:orange, *in-vivo*+*in-vitro*: red, *in-vivo*+*in-vitro*+*Y2H*:black. The dots representing the matrix entries have been enlarged for better visibility.

Figure 5 shows two representations of the adjacency matrix of the PIN. In panel A, the rows and columns are ordered according to the assignment of nodes into classes with the highest scoring non-diagonal image graph. In panel B, rows and columns are ordered according to the assignment of nodes in classes when fitting a diagonal image graph, *i.e.* when searching for cohesive modules. In both cases we allowed for 11 classes. The example allows us to highlight again the differences between a partition into cohesive modules and functional roles. Note the similarities and differences in the matrix when ordered after fitting a diagonal image graph and after fitting a non-diagonal image graph.

The non-diagonal models also allowed capturing groups of proteins, such as group 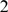, that mediate between cohesive clusters or that form a cohesive overlap between cohesive clusters, such as groups 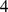 and 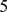 or 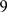 and 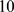.

When comparing the cohesive modules to the functional roles (Fig. 5) the most distinguishing feature is the existence of pronounced connections between sets of proteins in the latter. Groups of proteins exist, which all performed the same “functional role” of connecting two other groups of proteins. A separation of the cellular network into cohesive modules must necessarily omit these characteristics of the network. In the functional role model, groups are connected to other groups by a distinct set of additional proteins. These “connector groups” may themselves be cohesive, but do not have to be. This is illustrated by class 2, where most of the proteins are not interacting with other proteins in the class, but with those of groups 1 and 3.

To evaluate the biological significance of this result, we return to our GeneOntology enrichment analysis. Class 2 is significantly (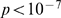) enriched in proteins annotated as belonging to the membrane and plasma membrane compartment. Indeed, this class contained many transmembrane proteins such as Cadherin. These proteins typically do not interact with many other transmembrane proteins, but rather with their extra-cellular binding partners and, in the case of transmembrane receptors, with cytoplasmic signal transmitters. Indeed, we found that group 1, highly interacting with proteins of class 2, mainly consists of proteins localized in the extracellular region (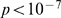). Furthermore, group 3, also strongly interacting with proteins of class 2, was enriched in proteins associated with the plasma membrane (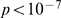) and involved in signal transduction (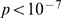). Thus, the transmembrane proteins of class 2 are the perfect biological implementation of proteins not interacting with each other, but instead with proteins of other classes (nodes of type 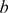 in figure 1 A). They could not be recognized by a method focusing on cohesive modules alone.

**Figure 6:**
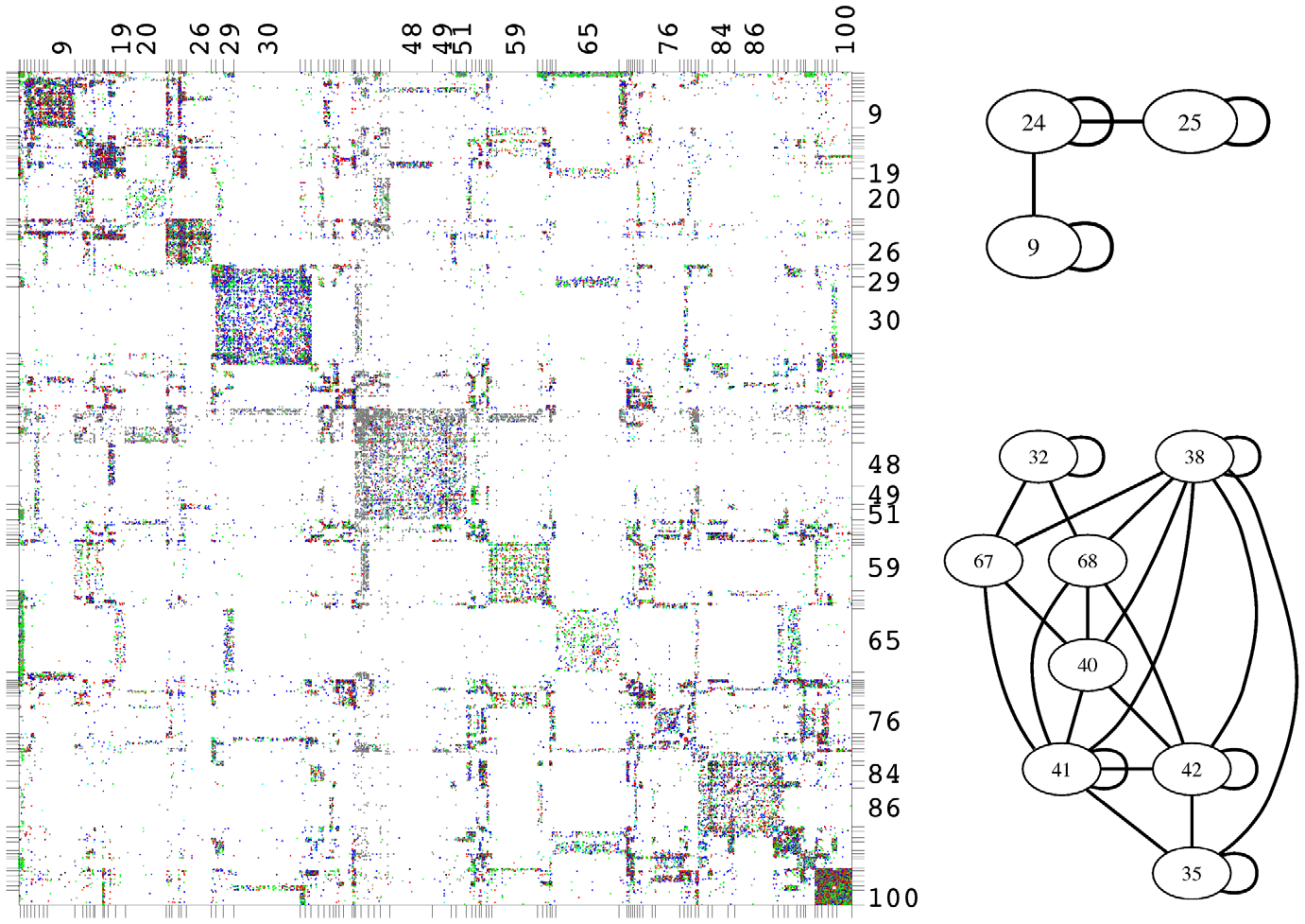
Block assignment in a functional role model with 100 classes. Adjacency matrix with rows and columns ordered according to assignment of proteins into classes. Color code and size of dots representing matrix entries as in figure 5. Only classes containing more than 100 proteins are labeled for better readability. Two details from the corresponding image graph exemplifying the kinds of structures found by the algorithm.

Next, we consider a non-diagonal model with 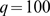 classes. Figure 6 shows the adjacency matrix of the network with rows and columns ordered according to the assignment found by the algorithm. The entire 100 node image graph is connected but too complex to be discussed within the scope of this paper. Instead, we focus on two small subgraphs, as shown in figure 6, which exemplify two typical substructures in the network that could not be discerned by methods focusing on cohesive modules alone, and discuss their biological interpretation in greater detail. Note that in figure 6 we only show the classes and links between classes discussed below and that the majority of classes shown have additional connections with the rest of the image graph.

As a first example, consider the proteins of the clusters 24 and 25. They form two cohesive modules which are also frequently interacting. Still, they are separated in two distinct groups. Inspection of interaction patters outside the main diagonal reveals that proteins in cluster 24 are frequently interacting with proteins in cluster 9, whereas proteins in cluster 24 do so only rarely. What could be the biological reason behind this pattern? Both clusters 24 and 25 are highly enriched in transcription factors. Their interaction is a typical feature in the regulation of transcription. Cluster 9, which distinguishes the two groups of transcription factors, shows an enrichment in proteins associated with ubiquitin-specific protease activity as well as polymerase activity. Indeed, ubiquitination plays a significant role in the regulation of transcription (for reviews see for example [39],[40]). Thus, our algorithm was able to detect structure even in highly connected sets of proteins (24+25) and to subdivide a group of highly interacting proteins by the presence and absence of interaction with other proteins outside of these clusters. Biologically, the transcription factors of cluster 25 are a good starting point for the further analysis of the role of ubiquitination in transcription regulation.

As a second example, consider the proteins in cluster 40. A method focussing on cohesive modules would not group these proteins together as they are hardly interacting with other proteins of the same cluster, but rather proteins in the “surrounding” clusters (38,41,42) as well as 67 and 68. According to the GeneOntology analysis, all of these clusters are enriched in proteins with a serine/threonine kinase activity. No functional enrichment, however, is found in cluster 40 itself. The only significant signal revealed that 64 of the 90 proteins in this cluster are localized in the cytoplasm. What kind of cytoplasmic proteins could interact with serine/threonine kinases, but not with other proteins of a similar interaction pattern? A manual inspection of the annotation of the proteins in cluster 40 found 12 proteins which are involved in the modification of non-protein substrates. These include glucose-6-phosphatase (NP_000142), 6-phosphofructo-2-kinase (NP_006203), but also adenylate cyclases (NP_001105, NP_001106). Thus, this cluster may consist of cytoplasmic proteins whose activity has to be tightly regulated by protein kinases but perform actions on non-protein molecules. This finding might help to elucidate the function of so far only cursory analyzed proteins within this cluster such as the “unnamed protein product” (BAC87492). Why were the regulating protein kinases put into different groups? As the algorithm considers the overall connective behavior of the proteins, the only difference could come from differing further clusters interacting with these proteins. Indeed, the clusters 67/68 are connected with cluster 32 but not with 35, whereas 41/42 are connected to 35 but not 32. Whereas cluster 32 contained mainly proteins associated with the plasma membrane, cluster 35 was enriched in nuclear proteins. Together, this reveals that the proteins in cluster 40 may be regulated by two types of protein kinases, which are localized in the nucleus and the cytoplasm, respectively.

This example, again, shows how putative protein functions may be inferred from the topology of the PIN. The consideration of classes of proteins with more diverse connectivity profiles than cohesive modules also allows for a more refined view of network topology and thus holds the promise for better protein function inference.

Complementary to the example with 11 classes, the 100 class model showed how our approach can be used to zoom in a top-down fashion into the architecture of a cell. Even at this finer-grained level, the whole network is considered, as the given examples illustrate. This can be seen as a major distinction from a protein centric view, which would cluster by a “guilt of association” approach. From the viewpoint of an experimentalist working with a few proteins, our clusters might be useful to find other proteins with a similar interaction behavior. Thereby, one might experimentally characterize specific regions of a network without losing the background of the cellular architecture.

#### Distribution of experiment-type in PIN

**Figure 7:**
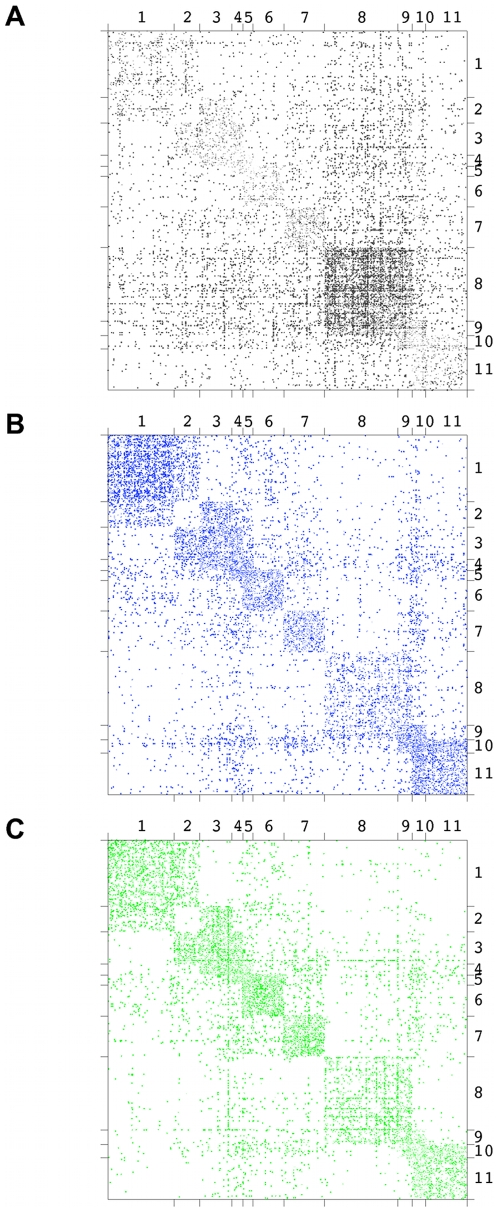
Comparison of block assignment. The same assignment of nodes into 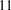 classes as used in figure 5 but for 3 different types of interactions, separately. A Interactions reported only for yeast-2-hybrid experiments (grey). B Interactions reported only in *in-vitro* experiments (blue). C Interactions reported only in *in-vivo* experiments (green). While *in-vitro* and *in-vivo* data is highly correlated, the interactions found in *Y2H* experiments are enriched in class 8.

Visual inspection of the adjacency matrices with the experiment type color coded as in fig 5 or 6 seems to suggest that interactions are not found uniformly distributed in blocks. In particular, *Y2H*-only backed interactions seem to be distributed differently than any other experiment type. To unravel a possible bias between different experimental methods, we plotted the data for three different experimental approaches separately. The ordering of rows and columns, *i.e.* the assignment of proteins into functional roles, was kept from figure 5. Instead of plotting all types of interactions on top of each other, the adjacency matrices for interactions which are backed by *in-vivo*, *in-vitro* and yeast-two-hybrid [41] (*Y2H*) experiments were shown separately (Fig. 7). The *in-vitro* and *in-vivo* data nicely resembled the overall picture while the *Y2H* data did not follow this pattern. Rather, the data based on yeast two hybrid showed a prevalence for class number 8 in figure 7. In this cluster nuclear proteins were significantly over-represented (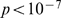). In the *Y2H* [42] assay, the tested proteins are fused to parts of a transcription factor. Their interaction is measured by the transcription of a reporter gene. Therefore, the proteins have to be within the nucleus. Thus, a bias towards interactions of proteins which naturally reside in the nucleus can be expected in *Y2H* data.

We now ask, whether we can show a systematic bias in *Y2H*-data in HPRD. So far, in the optimization of (5) and (4), we have considered all pairwise interactions between proteins in a weighted adjacency matrix. We assigned different weights for different experiment types reflecting a ranking of belief we have in the different data sources according to table 1. Interactions reported from *in-vivo*, in-vitro and *Y2H*-experiments were given the highest weight of 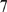, interactions reported only from *Y2H*-experiments only, were assigned the lowest weight of 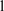. Since the quality functions (5) and (4) both normalize by the total sum of all weights, only the relative difference in weight is important. Consequently, when optimizing (5) and (4), interactions with a high weight will naturally have greater impact on the fit score and hence the optimization process will try to find assignments that give a particular good fit to the interactions with a high weight. This is desired as interactions backed by three experimental techniques are more likely to be correct and hence biologically relevant.

**Table 1:** Experiment type to link weight transformation.

Experiment type	Weight	# of interactions	distinct proteins involved
yeast 2-hybrid	1	6,580	3,727
in vitro	2	7,872	4,302
in vitro+yeast 2-hybrid	3	1,298	1,523
in vivo	4	6,721	3,826
in vivo+yeast 2-hybrid	5	824	1,119
in vitro+in vivo	6	6,877	3,781
in vitro+in vivo+yeast 2-hybrid	7	2,159	2,201

We valued the different experiments compiled in the HPRD database differently, giving lowest weight to interactions found in yeast-2-hybrid experiments only and highest to those interactions found in vivo, in vitro and *Y2H* experiments. These weights are only to represent a ranking of a practitioners belief in their validity.

Taking the assignment of proteins into cohesive clusters or functional roles as a results of our optimization on the full, weighted network and the resultant image graph, we can now easily calculate the fit score 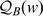 from (2) for each set of interactions corresponding to only one particular kind of experimental evidence. These scores are directly comparable even if the proportions of links backed by different experimental techniques are not equal as the calculation of 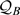 involves a normalization by the sum of edge weights. From the above discussion, we assume that the fit scores for each interaction type are an increasing function of the edge weight. We have already seen that the fit score is an increasing function for the number of allowed classes. In order to remove this latter dependency, we normalized the scores for the different edge types by the fit score of the entire network. These ratios of scores were then averaged over all values 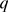 we considered in figure 3.

**Figure 8:**
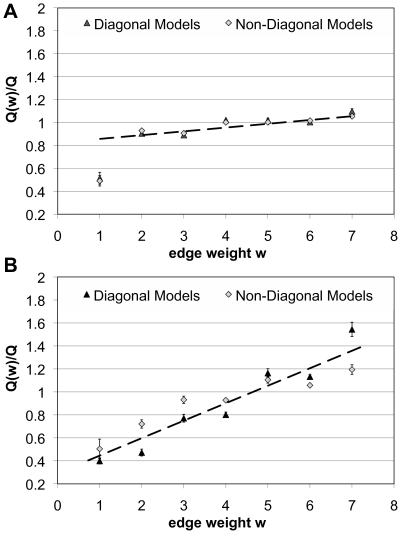
Fit-score as a function of link weight. Averaged over 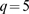 to 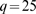, 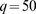 and 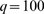, we show the fit scores 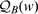 and 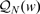 for each link type individually. Scores are normalized to the fit score obtained on the full data set from which also the assignment of nodes into classes and the image graphs are taken. A Actual HPRD data. Standard deviations are smaller than symbol sizes. B Randomized version of HPRD. As expected, we find the score increasing with weight. In the real data, increase of score with weight is slower, indicating a high correlation between the scores obtained for links representing different experimental techniques. As an exception, interactions with weight one, *i.e.* representing to *Y2H*-data only, show a significantly lower score than expected. See text for details.

Figure 8 shows these averages for the actual data in panel A and for the randomized data already used for figure 3 in panel B. Let us first focus on the results for the randomized data. We clearly see that links with a higher weight show higher fit scores. We further note a large variance of the scores around the linear trend. This is in fact a result of the kind of randomization chosen, which keeps the number of interactions *and* their types constant at each node in the network, because two links corresponding to different experimental evidence are never cross-wired. If we also randomize the types of interactions, which would correspond to rewiring all links in the network and then redistributing the weights randomly again, the curves smooth to a purely linear trend. Comparing the data on the randomized network with the actual data in panel A of figure 8, we first note that the slope of the increase in score with weight is much smaller. This is a clear indication that links corresponding to different experimental techniques are in fact highly correlated with respect to the block structure in the network. The randomization removes this very correlation and we thus observe the higher slope on the randomized data. Further, we note the small differences between diagonal and non-diagonal image graphs. This is due to the fact that, when averaged over the 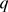 values used in figure 3, the difference in scores between diagonal and non-diagonal image graphs is relatively small. What is most striking to see is that only the scores for links with weight one, *i.e.* those interactions backed by *Y2H* evidence only, fall off drastically from this trend. We take this as a clear indication that the structural correlation between interactions found by *Y2H* experiments and other experimental techniques is low, and in particular, that we cannot expect *Y2H* data to cover the entire range of possible protein-protein interactions.

## Discussion

Using a suited algorithm, any network can be separated into cohesive groups of nodes with more internal than external connections. Accordingly, protein-protein interaction networks can also be divided into relatively independent units as putative functional modules [4]. Do these modules really reflect a typical characteristic of the cellular network? Here we used an alternative approach for the clustering of protein interactions. We grouped proteins of a similar functional role together. The functional role was defined by the interactions with proteins of other groups. In contrast to cohesive modules, which are more or less independent, groups which specifically linked other groups of proteins could be identified. Thus, an interconnectivity of biological units, as in the case of shared components in protein complexes, can also be observed at the cellular level. Using a GeneOntology based classification of all proteins within the modules, we found that these roles are mainly determined by cellular localization but also by function. Although possibly not too surprising to the biologist, this result underlines that the classes we identified by automatic clustering do represent a biological signal.

Using HPRD as a data source, a large-scale set of interactions with, on average, eight connections per protein could be analyzed. As HPRD contains manually curated data, their quality should be high enough to extend the results to higher coverage. The analysis of interactions derived by different experimental methods revealed a bias in the coverage, especially for yeast-two-hybrid data. The great difference of the protein interactions verified only by *Y2H* to the other methods reminds us to pay attention to the careful weighting of quality and quantity. As large scale binary interaction analyses were mainly based on *Y2H*, using high coverage data such as that from *Saccharomyces cerevisiae* or *Drosophila melanogaster* might even blur the signal. Another drawback was the small number of interactions per protein, around three or four for the yeast, fly and nematode sets analyzed in the study by Wang and Zhang [17]. Still, it would be interesting to compare networks between different organisms to see whether there are changes in the clusters correlated, for example, with the emergence of multicellularity. Contrasting to previous approaches, which compared networks either globally [43],[44] or locally [45],[46], comparing the image graphs allows detection of changes in the overall layout of the protein interaction network. But, reliable results can only be obtained when analyzing data sets of comparable quality and size [47].

In summary, our analysis showed that protein interaction networks are more than sparsely interacting cohesive modules. Rather, groups of proteins are connected by distinct sets of other proteins. These may be highly connected internally, but do not have to be. Therefore, functional roles and corresponding image graphs provide better descriptors for the characteristics of a protein interaction network than cohesive modules alone. They can help to further improve protein function prediction based on protein-interaction networks.

## Materials and Methods

### 

#### PIN network

We used the binary protein-protein interaction data from the HPRD [48] (Version 6). HPRD protein identifiers and experiment types used to support their connection were extracted. The experiment types were transformed to weights according to table 1. The analysis was restricted to the largest connected component containing 32,331(out of 34,367) interactions of 8,756 proteins (out of 8,919). These interactions do not include data inferred from protein complexes which may introduce errors and bias into the network structure [17].

#### Benchmarks

All algorithms were run on the same set of test networks. Each data point results from an average over 50 different realizations of the test network. For each test network, we chose the best of 10 runs starting from different random initial conditions according to the quality function associated with each algorithm, *i.e.* the highest 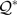 for our method, the highest log-likelihood for the Newman-Leicht method (NL) [30], the minimal description length for the Rosvall-Bergstrom Algorithm (InfoMod) [31], and the lowest Kullback-Leibler divergence for the non-negative matrix factorization (NMF). The multiplicative update rules for the NMF where derived as in [32], but for a symmetric tri-factorization as proposed by Ding *et al.* [33] from the following quality function:
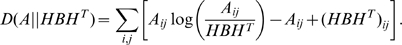
(6)Here 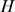 is an 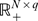 matrix and 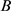 is an 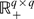 matrix. This particular form allows a direct assignment of the nodes into the class with the largest component in the corresponding row of 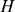. We found that 100 iterations of the update equations were enough to obtain convergence. For the NL method, which assigns class probabilities to each node, each node was assigned that class with the highest probability. The number of classes to detect is an input parameter for our method as well as for the NL and the NMF methods and was assumed to be given as 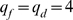. The InfoMod method explicitly infers the number of classes and thus is not provided with this input parameter.

Accuracy is measured via the normalized mutual information (NMI) introduced in Ref. [49]. It is based on the confusion matrix 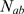 which measures how many nodes from designed class 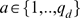 are found in class 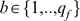 by the algorithm:
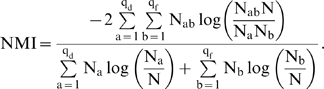
(7)where 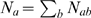 and 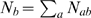 are the row and column sums of the confusion matrix.

#### Clustering

We optimized (5) and (2) using Simulated Annealing [29]. Details about the implementation can be found in [26] and [38], respectively. To obtain panel A of figure 3, for 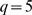 to 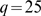, 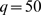 and 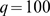 classes, we chose the best of 10 runs each, for both the fit of a diagonal block model as well as the detection of a non-diagonal block model. We employed a geometric cooling schedule. For fewer than ten classes we used a cooling factor of 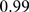 and one of 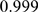 otherwise. On standard PCs, this led to runtimes between (minutes for the diagonal models with small numbers of classes) to days (for the non-diagonal models with large numbers of classes) due to the scaling of the runtime with the square of the number of classes.

#### Network randomization

In order to compare the fit scores for the real network with a randomized version of the HPRD database in figure 3 A, we used the rewiring algorithm of Maslov *et al.* [50]. This algorithm repeatedly and randomly selects two edges which do not share a node from the network, *e.g.* 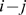 and 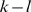, and rewires them as either 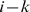, 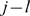 or as 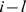, 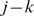, provided that non of these edges already exist in the network. This keeps the number of interactions constant for each node but removes all further structure. Since the PIN consists of several different types of links representing different experimental conditions under which the interactions were observed, we only rewired links of the the same type, thus keeping the number of interactions constant for each type at every node as well. The data points of figure 3 then represent averages over ten different realizations of a randomized network.

To obtain figure 3 B, we randomly divided the original set of links into a test and a training set of links. For fewer than 50 classes, the test set contained 1000 links and 100 otherwise. We used the image graphs, both diagonal and non-diagonal, found in the earlier experiment on the full data-set to optimize the fit score on the training-set. For less than 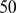 classes, the data shown are the fit scores of the test set, averaged over ten different partitions of the links into training- and test-set.

#### GO Term enrichment analysis

GO enrichment analysis was done using the “Ontologizer” by Grossmann *et al.* [37],[51]. It uses a modified Fisher's exact test and controls for the dependencies between terms introduced by the structure of the GeneOntology. The enrichment analysis for each class of proteins detected was done for this class with respect to the rest of the proteins in the network. The HPRD identifiers and their corresponding GO identifiers were taken from the same HPRD dataset as the protein-interaction network, re-formatted and saved into a file readable by the Ontologizer. For the Ontologizer the file gene_ontology.obo created by the GO project [52] was downloaded.
